# The silent companion

**DOI:** 10.1017/S1478951526101916

**Published:** 2026-02-23

**Authors:** Pankaj Kumar Garg

**Affiliations:** Department of Surgical Oncology, Shri Guru Ram Rai Institute of Medical and Health Sciences, Shri Guru Ram Rai University, Dehradun, India

Advances in oncology have transformed what we can offer patients – complex surgeries, refined radiation techniques, cytotoxic and targeted therapies, and immunotherapy. Yet, despite these advances, I have come to recognize that outcomes in cancer care are shaped not only by treatment protocols or technologies but also by forces that remain largely invisible in clinical discourse. Among the most powerful of these is the presence of a caregiver (Kaul and Garg 2023; Pandey et al. 2024).

This realization did not emerge from data or guidelines, but from a clinical encounter – one of many in everyday oncology practice – that was unusually pronounced and has stayed with me.

A woman once came to my outpatient clinic with her husband, a man with advanced oral cancer. He had already undergone extensive surgery followed by adjuvant chemoradiation. He now presented with a contralateral nodal recurrence that was unresectable and progressing despite palliative chemotherapy. From a biomedical standpoint, his disease had exhausted almost all standard options. He was frail, exhausted, and receiving best supportive care.

What struck me most, however, was not his condition, but hers.

She had traveled from a remote, mountainous region, carrying carefully organized files – histopathology reports, imaging, treatment summaries, and referral notes from multiple cancer centers. She listened attentively, rarely interrupting, absorbing each detail. Her fatigue was evident, but so was her resolve.

As I reviewed the records, it became clear that there was little more than conventional oncology could offer. After a pause, I asked her gently, “What do you expect from me?”

Her answer was immediate.

“Anything. I want to save his life.”

There was no negotiation, no visible doubt – only certainty.

I explained that immunotherapy remained a possible option, contingent on PD-L1 testing. I also explained the uncertainty of benefit, the financial implications, the need for repeated hospital visits every 3 weeks, and the physical and logistical demands this would impose. I asked how she planned to manage all of this.

“I will find a way,” she said quietly.

The tumor was tested. The PD-L1 score returned as 85%. Immunotherapy was initiated, alongside careful attention to symptom control, wound care, and nutrition. Over the following weeks, the tumor began to regress. His wound improved. His appetite returned. He started gaining weight. What had appeared to be an inexorable decline slowed, then partially reversed.

The clinical response was striking, even to us.

Yet, as the weeks passed, I found myself thinking less about the drug and more about the person who ensured that every appointment was kept, every dose administered, and every complication addressed. It was his wife who sustained the rhythm of care – managing pain medications, wound dressings, nutrition, travel, finances, and hope – often simultaneously.

In oncology, we are trained to measure responses: tumor size, survival curves, and toxicity grades. We document what can be quantified. But caregiving resists easy measurement. Its labor is emotional, logistical, and moral. It unfolds in waiting rooms, overnight wards, and long journeys home. It is rarely acknowledged, yet it shapes what patients can endure and what treatments remain possible.

This encounter forced me to confront the limits of our language in medicine. We speak fluently about disease, prognosis, and therapy, but far less about the quiet work that allows care to continue when certainty has disappeared. Caregivers often inhabit this space – absorbing uncertainty, sustaining hope, and carrying the weight of decisions long after clinical conversations end.

The man eventually stabilized for a meaningful period. Whether the outcome would have been the same without his caregiver’s unwavering presence is impossible to know. But what is clear is that her role was not ancillary. It was foundational.

As clinicians, we often stand at the center of the cancer narrative. This experience reminded me that, more often than not, we are accompanied by someone whose contribution is less visible but no less essential. The caregiver walks beside the patient through fear, fatigue, and fragile hope, long after our clinic doors close. Their labor – emotional, logistical, and moral – rarely finds space in clinical documentation, yet it quietly determines what care becomes possible (Applebaum and Breitbart 2013; Badr et al. 2014; Longacre et al. 2012).

While various national and thematic observances acknowledge caregiving in general, there is no dedicated global day recognizing family caregivers in cancer care. This reflection therefore proposes the idea of a “World Cancer Caregivers Day” – a symbolic moment devoted to acknowledging and honoring the families and loved ones who quietly sustain patients through the cancer journey. Such recognition would not alter disease biology or treatment algorithms, but it may help make visible a form of care that is foundational, enduring, and too often taken for granted.
